# Digital Strategy Decision Support Systems: Agrifood Supply Chain Management in SMEs

**DOI:** 10.3390/s22010274

**Published:** 2021-12-30

**Authors:** Maria Kamariotou, Fotis Kitsios, Chrysanthi Charatsari, Evagelos D. Lioutas, Michael A. Talias

**Affiliations:** 1 Department of Applied Informatics, University of Macedonia, 156 Egnatia Street, GR 54636 Thessaloniki, Greece; mkamariotou@uom.edu.gr; 2 School of Agriculture, Aristotle University of Thessaloniki, GR 54124 Thessaloniki, Greece; chcharat@agro.auth.gr; 3 Department of Supply Chain Management, International Hellenic University, PC 60100 Katerini, Greece; evagelos@agro.auth.gr; 4 Healthcare Management Postgraduate Program, Open University Cyprus, P.O. Box 12794, Nicosia 2252, Cyprus; michael.talias@ouc.ac.cy

**Keywords:** decision support systems, agrifood supply chains, information systems planning, digitalization, SMEs

## Abstract

The specific attributes of agrifood supply chains, along with their importance for the economy and society, have led to an increased interest in the parameters that enhance their effectiveness. Recently, numerous digital tools aimed at improving supply chain effectiveness have been developed. The majority of existing research focuses on optimizing individual processes rather than the overall growth of a food supply chain. This study aims to identify the stages of the information systems planning (ISP) process that affect the success of developing a strategic decision support system (DSS) for improving the decision-making process in the agrifood supply chains. Data were collected from 66 IT executives from Greek small and medium-sized enterprises (SMEs) in the agrifood sector and analyzed using regression analysis. The results revealed that situation analysis is the only stage of ISP that predicts ISP success. These findings can assist managers in appreciating the critical role of ISP for improving the performance of agrifood supply chain operations. Implementing the most appropriate information systems (IS) and digital tools results in increased competitive advantage, cost savings, and increased customer value.

## 1. Introduction

In the recent complex environment, firms must examine how to enhance the sustainability and performance of their supply chains. Digital tools can be used as they allow companies to share inventory and capacity plans, forecasts, financial data, databases, and data about products which improve the performance of supply chains. The use of digital tools help firms to become an important part of the supply chain as intermediaries or online e-marketplaces [1,2,3,4,5,6].

Small and medium-sized enterprises (SMEs) that operate in the agrifood sector must operate in a new and complicated technological environment. This challenge requires increased complexity and radical shifting. Furthermore, these circumstances may influence several business processes reduce their capacity to anticipate environmental uncertainty. Other factors that reduce their ability to anticipate environmental uncertainty other than financial difficulty, are the lack of human, technical, and administrative resources, as well as the lack of strategic planning [7,8,9]. Formal processes that are combined with strategic planning and data handling can support managers to increase business performance.

Scholars have paid attention to supply chain effectiveness especially in the agrifood industry. Although, the agrifood sector is a significant pillar of economic development, academics have not focused on the area of food supply chains in the agrifood industry due to the unique attributes of its products. Companies from different sectors must collaborate to deliver products to the market and meet consumer needs because food supply chains are complex [9,10,11,12]. However, firms that operate within this sector ignore the significance of aligning digital tools with their current business processes. Therefore, limited technological tools can support executives to handle data and make effective decisions. A fast supply chain is needed in the agrifood sector as products have specific attributes. Therefore, executives have to develop digital tools and systems that improve the effectiveness and performance of supply chains [13].

Agrifood supply chains (ASCs) support the flow of data and products between suppliers and customers. Nevertheless, supply chain constraints have created several operational barriers making collaboration among farmers, retailers, food manufacturers, and customers difficult. These obstacles have increased because small farmers do not have the resources to concentrate on logistics, transportation, warehousing, and marketing [14,15]. 

Agrifood supply chains frequently do not develop in a strategic manner meaning that executives cannot formulate strategies that will strengthen long-term relationships with their suppliers and consumers. As the complexity of the external environment has increased, an efficient and timely decision-making process is necessary so that organizational goals and supply chain strategies can be achieved [16,17,18,19,20]. Existing studies [21,22] highlight that SMEs have already adopted and used digital tools to integrate technology into business processes. Both scholars and practitioners have focused on the strategic planning of IS to help executives integrate digital tools into agrifood supply chain processes and support them to enhance the effectiveness of decisions. 

Thus, this article aims to explore the stages of the Information Systems Planning (ISP) process that affect the success of the development of a strategic Decision Support System (DSS) for effective decision making in agrifood supply chains (ASCs.) 

The structure of the article is the following. Section 2 outlines the theoretical background, also presenting existing DSS models that have been developed for ASCs. The Section 3 describes the methodology and Section 4 presents the findings of this study, which are discussed in the Section 5. Finally, the paper closes with concluding remarks (Section 6).

## 2. Theoretical Background

### 2.1. Agrifood Supply Chains

Supply chains are networks of organizations that engage in upstream and downstream processes and enact specific activities with the aim of producing value for both the entities involved in the chain and the final consumers, in the form of products or services [23]. The sourcing of raw materials, the processes of manufacturing, assembly, warehousing operations, inventory and order management, the distribution procedure across the chain, the delivery to the end consumer, and also the flow of information and capital within the network, are some of the activities linked to supply chain management [24,25]. Although the term “chain” denotes a linear configuration resembling a pipeline structure, through which products are transformed into final goods and delivered to consumers, supply chains are in fact complex networks, connected with external and—sometimes—loosely linked actors, and exposed to the wider economic, social, and technological environment [26,27].

ASCs are those supply chains that aim at the movement of agrifood products from production to consumption, including pre-production practices and post-consumption activities [28]. As in the case of industrial supply chains, ASCs are open systems, characterized by the existence of different subsystems and emergent properties, also being vulnerable to the external environment [29,30]. However, such supply chains have particular characteristics that affect their modus operandi. 

First, the nature of agrifood products heavily impacts the operation of an ASC. The perishability of production, along with specific and long production cycles, seasonality, and uncertain quality and quantity of production due to climate conditions or plant/animal diseases [31] make the formulation and the implementation of any strategy difficult. In addition, the perishability of products eliminates the possibility of keeping buffer stocks, thus challenging the (vertical) coordination of ASCs [32].

Second, the structure of markets and the power hierarchies it builds greatly influence the functioning of supply chains. Although food systems and associated ASCs are not uniform around the world [33], evidence suggests that agrifood markets are highly concentrated, with leading retail and processing companies representing dominant players in the global agrifood system [34,35,36]. Concentration generates oligopsonistic conditions [32], creating complex forms of dependence among the actors involved in ASCs.

Third, the actors involved in ASCs have to cope with changing consumer life-styles and preferences [37,38]. Much more than other types of products, agrifood commodities face changing demand patterns that create fluctuations in supply chain operations. Increasing concerns over food safety [39], the processes of food production [40] and the technologies used [41], the substances added to food products [42], and the environmental, social [43], and ethical [44] dimensions of agrifood goods lead companies involved in ASCs to constantly redefine their purpose and values, and to modify their strategies accordingly. The shift of relevant policies towards more healthy and environmentally friendly food [45,46] further increases the need for companies to continuously adapt their strategies as a means to sustain their market position.

### 2.2. Decision Support Systems in Agrifood Supply Chains

Fanti et al. (2015) [47] presented a DSS model which pays attention to the assessment of transportation performance. A database gathers information regarding product and service prices, budget allocation, resources, and costs, and then performance indicators are calculated using simulations about transportation. Songbai et al. (2010) [48] developed a DSS for vehicle routing. This system analyzes information regarding demand, the strength of the vehicle, the number of drivers, and mileage per vehicle. Managers can use this system to make operational decisions about transportation personnel requirements, appropriate routes, and vehicle demands based on optimization methods. Kengpol (2008) [49] presented a DSS model for a logistics distribution network. The system analyzes data regarding locations, customers, and transportation costs to develop alternative solutions and assess them. 

Existing DSS ignore important tasks of strategic planning such as the definition of objectives, the analysis of the internal and external environment, and the implementation and assessment of the supply chain’s strategy. In addition, existing systems in supply chains have paid attention to the technical characteristics of collecting, visualizing and assessing data, ignoring strategic aspects. Validi et al. (2014) [50] proposed a DSS for coordinated distribution systems. The purpose of this model was to increase the effectiveness of logistics and reduce environmental impact. Techniques about location routing visualization were implemented to minimize costs in the supply chain. Other scholars have focused on crop production systems to support managers to handle information regarding production costs, availability of land and water, and uncertain labor supply. The creation and evaluation of feasible crop rotations on a vegetable farm was implemented using linear programming and network flows [51,52]. Lao et al. (2010) [53] developed an integrative food handling system and a warehouse system. However, they concentrated on the technical characteristics of the system. Allaoui et al. (2018) [10] and Brulard et al. (2019) [54] concluded that the development of a comprehensive model is required which will focus on specific objectives and indicators to assess supply chain performance and support strategic and tactical decisions. 

### 2.3. Strategic Decision Support Systems in Agrifood Supply Chain

The ISP process can be used to develop a DSS for ASCs. The ISP process includes five phases. During the strategic awareness phase, which is the first phase of the ISP process, tasks concerning the determination of important planning issues, priorities, goals, and the selection of employees who will take part in the planning team of the process, are included. The second phase, the situation and significant risk analysis, includes the following: analysis of the existing business structure, analysis of existing organizational processes and systems, and analysis of the external and internal technological environment. During the third phase of the ISP process, IS managers identify important goals, opportunities for change, and high-level IS strategies. Strategy formulation is the fourth phase of the ISP process. The most significant tasks involved in strategy formulation are the following: the determination of new business processes and IT architecture to achieve IS goals and the definition of new IS plans and priorities that will support the performance of the firm. Finally, strategy implementation involves the determination of change management processes and action plans. In addition, in this stage, IS executives evaluate the output of the ISP process and examine if the objectives have been achieved [7,55,56,57,58,59,60,61,62,63,64,65,66,67,68,69].

According to the phases of the ISP process, the suggested strategic DSS model includes five stages. Strategic awareness entails identifying critical future challenges, objectives, and priorities, as well as selecting employees to serve on the DSS development team. These goals refer to harvesting, warehousing, customer service, transportation, food production, inventory management, and order processing. The following are the significant risks of the second stage, known as situation analysis: analysis of the existing business structure, analysis of existing organizational processes and systems, and analysis of the external and internal technological environment. Concerning the internal environment, executives examine strengths and weaknesses regarding production costs, harvesting policies, logistical costs, logistical structure, level of demand, inventory management, warehousing, transportation, prices, systems, and materials handling [67].

The analysis of opportunities of, and threats to, the business environment is necessary since companies operating in the agrifood industry are highly interdependent. Furthermore, this analysis supports the growth of the supply chains’ sustainability. An awareness of developments in business partner organizations, competitors, products, and markets is crucial to improving the supply chain’s performance. This analysis can be conducted using systematic scanning and through the relationship with business partners [12]. Other factors that affect this analysis are pressure from resource scarcity, competitors, consumer demand, isomorphism, and deregulation [14]. Therefore, managers should be aware of these factors to develop a DSS that will improve the performance of supply chains.

Moreover, managers require data regarding distribution channels, market segments where competitors are active, demands relating to product attributes, quality of suppliers, economic situation of suppliers, and buying power [12,70]. In addition, information regarding food production, healthy eating, the rural economy, the environment, and consumer values is also important [31]. Nevertheless, decision makers can analyze data monitoring competitiveness in the agrifood industry [12].

In the next stage, strategy conception, a database, application programs, and a data model are involved. This stage, which interacts with other stages, can use the results of the previous two stages as input. Thus, executives can collect, store, and retrieve the required data regarding external and internal environments as well as historical data in order to develop alternative solutions. Executives can then assess the data and choose the best alternative to develop further. These alternative scenarios regarding responsiveness, material flow, agility, costs, food quality, efficiency, and sustainability of supply chain are the outputs of this stage [71]. Mathematical models are used to develop alternative scenarios based on the problem which has been defined. Furthermore, many models, theories, methods, algorithms, and techniques, such as intelligent data analysis, optimization techniques, multicriteria methods, and fuzzy theory are used to analyze alternative scenarios [72].

The next stage of the DSS model is strategy formulation. The significant tasks involved in strategy formulation are the following: identification of new business processes and IT architectures to achieve the supply chain’s goals, and the definition of new IS plans and priorities that will support the performance of ASCs. Finally, strategy implementation involves the determination of change management processes and action plans. In addition, at this stage, IT managers assess the output of the ISP process and examine if the goals have been achieved.

What has been indicated by surveys examining the effect of ISP process on success is that IS managers have focused on strategic conception. Combined with opportunity analysis and evaluation, the strategy’s conception could offer more realistic alternatives. Understanding IS objectives can enable the company to define future IS and business goals. Better options and choices can be defined to produce better outcomes. The frequently encountered challenges that emerged during the execution of the ISP process were the lack of top managers’ engagement and the inability to develop effective action strategies to develop IT projects. If executives do not support the development of IS plans, team members will not be focused on the plans and will have difficulties implementing the IT strategy. Thus, it is preferable for managers to define the priorities that support their IT strategy to be better executed and achieve their objectives. Previous researchers have indicated that IT managers tend to pay attention to IT strategy implementation because they consider the execution of strategy to be a complex process [57,73].

Findings also indicate that some managers are overworked with respect to the ISP process whilst others are doing too little. Such approaches may prove ineffective. In the first case, the ISP process could be misunderstood, postponed, or stopped from being enforced, while in the second approach the implementation plans could be unsuccessful, meaning that their objectives could not be accomplished. The evaluation of the process is obviously of great importance if managers wish to minimize these unsatisfactory outcomes. Researchers have indicated that IT managers pay attention to strategy conception and strategy implementation, ignoring the significance of strategic awareness and situation analysis. As a consequence, the IT strategy which is being developed is not efficient and effective and it does not meet IT goals [74,75,76,77]. Furthermore, IT executives focus on reducing the required time and cost for the project. Executives pay attention to process implementation and this fact has negative results. Nevertheless, it reduces the time it takes for ISP process implementation, but the organization’s strategic goals are not aligned with IS objectives [20,78,79,80].

Regarding the existing literature five hypotheses have been identified:

**Hypotheses** **1** **(H1).** *Strategic awareness for the development of DSS positively affects ISP success in the agrifood sector.*

**Hypotheses** **2** **(H2).** *Situation analysis for the development of DSS positively affects ISP success in the agrifood sector.*

**Hypotheses** **3** **(H3).** *Strategy conception for the development of DSS positively affects ISP success in the agrifood sector.*

**Hypotheses** **4** **(H4).** *Strategy formulation for the development of DSS positively affects ISP success in the agrifood sector.*

**Hypotheses** **5** **(H5).** *Strategy implementation for the development of DSS positively affects ISP success in the agrifood sector.*

## 3. Methodology

To test the above-mentioned hypotheses, a quantitative study was conducted. A questionnaire was developed incorporating items, questions, and items used in previous studies examining the stages of the ISP process and their contribution to success [7,55,58,59,62,63,64,65]. The items referring to the five ISP stages/activities are presented in Appendix A. For all the items a five-point Likert-type scale was used.

Four IT managers participated in a pilot survey to provide feedback on the content of the questionnaire. After that phase, the questionnaire was administered to a sample consisting of IT managers from Greek SMEs in the agrifood sector [7,55,57,59,62]. The sampling frame consisted of IT managers who worked in SMEs that operate in the agrifood sector (located in the regions of Thessaloniki and Athens). The inclusion criteria were the number of employees (between 20 and 50) and the annual turnover (below 50 million euros). In total, 440 companies met these criteria. The authors contacted IT managers in these companies and invited them to participate in the study. The questionnaire was emailed (along with a cover letter) to those managers who agreed to offer data. After a short period, 66 respondents working in companies with an average turnover of three to ten million euros with 20 to 40 employees returned completed questionnaires. Data were analyzed using regression analysis.

## 4. Results

Table 1 presents details about the respondents and Table 2 presents details about the SMEs. Overall, 43.94% held a college degree while 40.90% had completed an advanced degree. Regarding IS experience, 37.88% had an average of 11 years’ IS experience while 34.85% had an average of 21 years’ IS experience. The average number of IS employees was 2 and the majority of SMEs had turnover between 11 and 50 million euros.

The reliability of variables was evaluated using Cronbach’s alpha and the values ranged from 0.899 to 0.912, exceeding the minimally recommended level of 0.70 [57]. Table 3 presents the Cronbach a value for each variable.

Pearson’s correlation was computed to explore the relationship among the study variables. The values of Pearson’s r are presented in Table 4.

ISP success is the dependent variable of the model. The values of R^2^ and adjusted R^2^ indexes are presented in Table 5. Based on these values, 68% of the variance in the dependent variable of the model is explained by independent variables. The value of the F statistic is 26.605 and the degrees of freedom are 66 (5 from the regression and 61 from residuals). As the significance value is less than *p* < 0.05 (0.000), we can conclude that the model sufficiently describes the data. Table 6 summarizes the findings of ANOVA statistics. These results also confirm the satisfactory predictive performance of the model. 

Based on the findings displayed in Table 7, situation analysis is the only contributing stage for ISP Success. The beta value of situation analysis is 0.260 with significance level 0.022. Therefore, situation analysis has a positive and significant impact on ISP success and H2 was supported. On the other hand, strategic awareness, strategy conception, strategy formulation and strategy implementation have a positive but not significant effect on ISP success. Thus, H1, H3, H4 and H5 were not supported.

## 5. Discussion

The results of this study indicate that executives in SMEs that operate within the agrifood sector associate situation analysis with the success of ISP. Furthermore, IT managers do not concentrate on strategic awareness. As a result, the outcome of the implementation of ISP processes is the development of inefficient IS and digital tools that cannot meet the agrifood supply chain’s objectives. The available budget for IS projects is often limited. Thus, managers do not focus on the definition of strategic goals such as how digital tools will improve the supply chain’s effectiveness. Instead, they overemphasize attempts to reduce the time and cost of the development of IS plans. As a result, IS plans fail to support companies to meet customers’ needs, align the developed systems with the existing ones, and increase the system’s flexibility without strategic planning. This observation led to the rejection of H5 [7,55,63,64].

Selecting team members to participate in the development of the IS plan is another fundamental task in the ISP process, but managers tend to overlook it. The importance of this task stems from the fact that team members can collaborate and develop skills to develop efficient digital projects. Therefore, executives should support employees during the development of IS plans to help companies achieve their supply chain objectives, improve business operations and firm performance. In addition, managers ignore identifying priorities, enhancing collaboration among employees, and providing guidance to increase the efficiency of IT projects and align them with organizational goals. Therefore, the findings of this study explain the rejection of the H1 and H3.

Executives focus their efforts on ISP process implementation but this phenomenon has significant barriers. Although less time may be spent on the implementation of the ISP process, the strategic goals of the supply chain might not be aligned with IT goals. Considering this challenge, academics [7,55] have concluded that changes in the internal environment of the organization increase uncertainty and change the contribution of digital tools to organizational processes. Thus, managers should take into consideration environmental scanning and the use of digital tools to align the digital projects of the organization with supply chain performance. These findings confirm the high importance that strategy formulation plays within ISP processes which can explain the rejection of the H4 [66,67,68].

The results presented herein indicate that when executives concentrate on situation analysis, the agility of strategy conception and strategy implementation will be increased. Executives can analyze existing business systems, the digital tools and both the organizational and the external technological environment to align IT strategy with the supply chain strategy. Considering this analysis, the developed IT plan will be remarkably enhanced with the exception of the required time and cost for the process. When managers are aware of the business environment, they can define crucial IS goals and opportunities to improve the supply chain’s effectiveness. Furthermore, they can assess these goals to identify high-level IS strategies during strategy conception [46,47,48].

All stages of the ISP process did not influence the four dimensions of success because managers in this sector often lack appropriate skills, they may be isolated and without prior experience or training in IS. Furthermore, other factors that prevent managers from engagement with ISP-related activities are age, the organizational culture of the company, and a lack of sufficient budget for IS projects. Thus, managers face difficulties understanding the significance of IS implementation and, as a result, face difficulties formulating, implementing, and evaluating strategic plans. Therefore, they ignore many stages of the ISP process, they do not support IT projects and, due to limited resources and lack of an innovation culture, they do not invest in IS.

## 6. Conclusions

This paper has examined the stages of the ISP process that influence the successful development of strategic DSS models which provide guidelines for effective decision making in the agrifood industry’s supply chain. The findings of this article highlight that the execution of the ISP process is a challenge for managers. Executives should understand the agrifood supply chain’s peculiarities, objectives, and supply chain strategies, as companies have many planning aspects to deal with. Thus, SMEs are should focus on all stages of the ISP process during implementation, so as to successfully develop a strategic DSS.

This article contributes to the existing literature on the digitalization of ASCs [81,82,83,84] by highlighting the importance of the strategic use of DSS models that will support the performance of ASCs. If managers understand the stages of the ISP process, they will not ignore the activities at each stage. By understanding the stages of the ISP process, managers will pay attention to the agrifood supply chain’s objectives and recognize the significance of the ISP process to their organization. Therefore, IS projects will have fewer problems, their quality will be improved and the rate of success for the ISP process will be enhanced.

This article has a practical contribution because the model can be considered an efficient strategic model which helps executives make more timely decisions regarding strategic and tactical issues. Existing systems have been developed for specific tasks such as vehicle routing, transportation, and the logistics distribution network. The suggested model is based on the ISP process of strategic DSS. The determination of objectives, the analysis of the external and internal business and technological environment, the organization of the development team, the assessment of opportunities, the enhancement of organizational processes and the evaluation of the ISP process are important stages when executives formulate IS strategic plans for the development of DSS, and managers should consider them.

Furthermore, the DSS model provides timely information to executives to help them analyze the business and IT environment. Thus, both the complexity of the environment and the risk under dynamic change are minimized. Another advantage of the DSS model is that it provides managers with the opportunity to evaluate the process to determine if the supply chain’s objectives are being achieved. If this is not the case, remedial action should be implemented to adjust the tasks that have been implemented or even to change the strategy itself. Finally, the system involves the participation of different levels of executives who strengthen the use of the DSS and decision-making effectiveness.

A limitation of this article is that the survey was only conducted with Greek SMEs. Further research could be implemented to broaden the sample and compare the findings of this article to those of other companies that operate in different countries. Implementing semi-structured follow-up interviews with managers is another avenue for further research which could provide meaningful insights. Specifically, with the use of semi-structured interviews, future researchers can make open discussions regarding the effect of ISP stages on success. By exploring IS managers’ perceptions about the ISP process, scholars can determine how the ISP process can be improved and which factors need attention during the implementation of IT plans. In addition, interviews can be conducted with farmers, product manufacturers, and retailers to take into consideration the indices that are necessary for the assessment of the IT strategy. 

The planning of DSS is based on the requirements for data of the current organizational operations. Future DSS models that will be developed will involve digital tools that depend on environmental changes and a company’s data requirements. These DSS models will assist managers in adapting their working practices in order to meet future expectations. This cooperation during the ISP process can increase adaptability and enhance congruence between strategic IT planning and market requirements. Therefore, future researchers can examine the challenges that arise regarding the collaboration between managers during the ISP process.

## Figures and Tables

**Table 1:** Respondents’ education level, age, and IS experience.

Education Level	Respondents	Percentage
Some college	10	15.16
4-year college graduate	29	43.94
Postgraduate degree	27	40.90
Total	66	100.00
**Age**	**Respondents**	**Percentage**
18–25	2	3.03
26–35	19	28.79
36–45	26	39.39
46–55	12	18.19
>56	7	10.60
Total	66	100.00
**IS Experience**	**Respondents**	**Percentage**
0–5	6	9.09
6–15	25	37.88
16–25	23	34.85
26–35	9	13.64
>36	3	4.54
Total	66	100.00

**Table 2:** Employees, IS employees, and turnover.

Employees	Respondents	Percentage
20–49	66	100.00
Total	66	100.00
**IS Employees**	**Respondents**	**Percentage**
0–5	61	92.42
6–10	4	6.06
11–20	0	0.00
21–30	1	1.52
31–40	0	0.00
41–50	0	0.00
Total	66	100.00
**Turnover**	**Respondents**	**Percentage**
<2 million euros	7	10.60
3–10 million euros	23	34.85
11–50 million euros	36	54.55
Total	66	100.00

**Table 3:** Reliability of variables.

Variables	Cronbach a Value
(1st stage)	0.900
(2nd stage)	0.905
(3rd stage)	0.907
(4th stage)	0.912
(5th stage)	0.904
Success	0.899

**Table 4:** Correlation analysis.

	(1st Stage)	(2nd Stage)	(3rd Stage)	(4th Stage)	(5th Stage)	Success
(1st stage)	1	0.687	0.691	0.600	0.717	0.717
(2nd stage)	0.687	1	0.611	0.592	0.685	0.725
(3rd stage)	0.691	0.611	1	0.626	0.622	0.681
(4th stage)	0.600	0.592	0.626	1	0.614	0.663
(5th stage)	0.717	0.685	0.622	0.614	1	0.720

**Table 5:** R^2^ values, estimate standard error, and Durbin-Watson statistic for the regression model.

R	R^2^	Adjusted R^2^	Estimate Standard Error	Durbin-Watson
0.830	0.689	0.663	0.350	2.017

**Table 6:** ANOVA statistics of regression.

Model		Sum of Square	Df	Mean Square	F	Sig.
1	Regression	16.359	5	3.272	26.605	0.000
	Residual	7.379	61	0.123		
	Total	23.738	66			

**Table 7:** Hypothesis testing.

Model	β	t-Value	Sig.	VIF
(1st stage)	0.168	1.390	0.170	2.824
(2nd stage)	0.260	2.354	0.022	2.355
(3rd stage)	0.165	1.510	0.136	2.297
(4th stage)	0.175	1.726	0.089	1.993
(5th stage)	0.211	1.823	0.073	2.584

## Data Availability

Data sharing not applicable.

## Appendix A

**Table A1:** ISP stages and activities.

Strategy Awareness (1st Stage)	Situation Analysis (2nd Stage)	Strategy Conception (3rd Stage)	Strategy Formulation (4th Stage)	Strategy Implementation (5th Stage)
Defining important issues about ISP	Analyzing existing business systems	Defining important IT goals	Defining new business processes	Identifying change management processes
Determining the goals of ISP process	Analyzing existing organizational systems	Defining opportunities to improve processes	Defining new IT architectures	Identifying action plans
Organizing the planning team	Analyzing existing IS	Assessing opportunities to improve processes	Defining specialized new IT projects	Assessing action plans
Obtaining willingness of top managers to be part of the process	Analyzing the existing external business environment	Defining high level IT strategies	Defining priorities for new IT projects	Identifying control processes
	Analyzing the existing external IT environment			

**Table A2:** Success dimensions and variables.

Alignment	Analysis	Cooperation	Capabilities
Top managers understood that IS improve business strategy	Opportunities for improvement in organizational processes improvement were defined	Unambiguous guidelines of managerial responsibility were developed to implement ISP	Ability to define important negative results
Understanding the strategic priorities of top managers	Managers changed organizational processes and procedures	Potential sources of resistance to IT projects were defined and solved	Ability to deal with surprises and crises
Defining opportunities about IT in order to help the strategic direction of the company	New ideas were developed to reframe organizational processes using IT	Open lines of communication with other departments were created	Ability to deal with unanticipated changes
IS strategies were aligned with the strategic plan of the company	Information needs of subunits were understood	The development efforts of many organizational subunits were coordinated	Ability to increase collaboration among members of the development team
IS objectives were adapted to change organizational goals	Managers understood the dispersion of information, applications, and other technical infrastructure used in the company	A uniform basis to set priorities was established	
Top managers were educated about the significance of IS	A ‘‘blueprint’’ was developed to define business processes	An increased level of agreement about the risks/tradeoffs among IT plans was achieved	
IT was adapted to strategic change	Increased comprehension of how the company actually operates	The overlapping development of significant systems was decreased	
The strategic significance of IT was evaluated	Business needs and the capability of IT to achieve certain requirements were evaluated		
